# Characteristics Associated With the Use of the Mindfulness Meditation App Headspace in a Large Public Health Deployment: Cross-Sectional Survey Study

**DOI:** 10.2196/73457

**Published:** 2025-08-22

**Authors:** Judith Borghouts, Elizabeth V Eikey, Cinthia De Leon, Stephen M Schueller, Margaret Schneider, Nicole A Stadnick, Kai Zheng, Dana B Mukamel, Dara H Sorkin

**Affiliations:** 1 Department of Medicine University of California, Irvine Irvine United States; 2 Herbert Wertheim School of Public Health and Human Longevity Science University of California, San Diego San Diego United States; 3 The Design Lab University of California, San Diego San Diego United States; 4 Department of Informatics University of California, Irvine Irvine United States; 5 Department of Psychology University of California, Irvine Irvine United States; 6 Department of Public Health University of California, Irvine Irvine United States; 7 Department of Psychiatry University of California, San Diego San Diego United States; 8 Dissemination and Implementation Science Center UC San Diego Altman Clinical and Translational Research Institute La Jolla, CA United States; 9 Child and Adolescent Services Research Center San Diego, CA United States

**Keywords:** Headspace, mental health, mindfulness, mobile health, mHealth, real-world use

## Abstract

**Background:**

Mindfulness-based apps can be an effective and accessible resource for mental health support. However, little is known about their use outside of research settings and what user characteristics relate to app use.

**Objective:**

This study aimed to examine the characteristics of people who decided to use, not use, or stop using Headspace within the context of a large-scale public deployment, which offered the mindfulness meditation app Headspace as a free mental health resource to community members.

**Methods:**

Nearly 100,000 community members received Headspace. All members (N=92,311) received an email inviting them to complete a voluntary and uncompensated survey. In total, 2725 participants completed the survey. The 20-minute survey asked about the use of Headspace, user experience, mental health problems, mental health stigma, and mental health use. Logistic regression models were used to examine relationships between predictors and nonuse, past use, or current use of Headspace.

**Results:**

Participants who were still using Headspace at the time of completing the survey (2076/2725, 76.18%) were more likely to experience mental health challenges and distress and make more use of other digital mental health resources (ie, online tools and connecting with people online) than people who were not using Headspace. In addition, current users of Headspace rated the app higher on user experience compared with past users. The most common reasons for abandoning Headspace were that people were already using other strategies to support their mental health (198/570, 34.7%), no longer needed Headspace (73/570, 12.8%), or did not think Headspace was useful (46/570, 8.1%).

**Conclusions:**

Results indicate that a person’s mental health challenges, a perceived need for support, and familiarity with digital resources were associated with continued use of Headspace. While the most common reason for not using Headspace was that people were already using other resources, it is important to consider the continuity of mental health support beyond these free programs for those who may not have easy access to other resources*.* We discuss potential implications of our findings for offering and using apps such as Headspace as a mental health resource, along with factors that influence engagement with this app.

## Introduction

### Background

Mental health is an increasing societal concern. The Centers for Disease Control and Prevention, for example, has formally recognized mental and psychological health as a major and global public health issue [1]. Around 1 in 5 adults in the United States are estimated to experience a mental illness every year, and a 2022 survey found that 90% of Americans believe that the United States is facing a mental health crisis [2].

Digital media can considerably increase access to mental health care. The development of digital mental health interventions (DMHIs) has accelerated in recent years due to a desire to find more scalable and cost-effective options that can overcome access barriers to care [3]. Examples of digital mental health mediums include internet-based therapy, as well as self-guided platforms such as websites and apps that provide psychoeducation, journaling tools, peer support, and mindfulness meditation. Mindfulness is a state of bringing nonjudgmental attention to the present experience. Mindfulness meditation is a practice that sets aside time to develop mindfulness, and mindfulness mediation apps tend to be some of the most popular and commonly downloaded DMHIs [4]. An analysis of 578 DMHIs found that mindfulness was the third most common feature, behind only psychoeducation and goal setting and habit, with 38% of products containing mindfulness features [5]. Systematic analyses and reviews showed that mindfulness meditation can have several mental health benefits [6,7]. Benefits include reducing stress, anxiety [8-10], depression [11,12], and psychological distress [13]. Thus, better understanding aspects about mindfulness meditation DMHIs provides useful information to the field.

DMHIs are beginning to be used as broad-scale supports for mental health, especially from a public health perspective where they can be made widely available. To increase awareness of and access to DMHIs, various initiatives by schools, workplaces, and other organizations have offered DMHIs to their community members free of charge [14,15]. DMHI companies themselves have also made their products available to specific populations or the public in times of need. For example, during the COVID-19 pandemic, some products were made freely available to help people cope with mental health concerns [16]. However, little is known about the characteristics of those who make use of these initiatives, as well as information about their engagement and experience with the DMHIs offered. In addition, few data are available about free-range, naturalistic DMHI use outside of research contexts. Research studies often provide compensation to complete study procedures, which may also impact use and engagement with DMHIs. Although these studies can demonstrate the potential and effectiveness of DMHIs [17,18], their effectiveness is ultimately related to their use, and previous studies researching mindfulness apps found a clear relationship between app use and improvements in mindfulness [6,19]. For instance, past research studies focusing specifically on the mindfulness meditation app Headspace with relatively small sample sizes [6,9] showed that Headspace was effective in improving depressive symptoms [9] and psychological distress [20] but only among study participants who used the app frequently. In addition, study participants received the app for free and were given recurring reminders to use the app [9] or daily surveys about their use of the app [20], which may have impacted engagement.

Engagement in real-world studies has shown considerable variation [21,22]. One study found that when metrics were available, research use was more than 4 times higher than real-world use across the same DMHI programs [23]. Unfortunately, data from broad deployments and real-world contexts are rarely available for others to learn from.

### Objectives

This study draws from a California-based innovation program which offered the mindfulness meditation app Headspace for free to community members as a mental health resource. The evaluation of this program provides a unique opportunity to understand factors in the context of real-world use rather than a research environment. The aim of the study was to understand the characteristics of people who decide to use, stop using, or not use an app such as Headspace within the context of the program, as well as their experience with the app.

Gathering data in real-world deployments often requires balancing the views and priorities of various stakeholders. In our study, we worked closely with community members from participating counties and cities (referred to as sites throughout the rest of the paper) to develop the survey and select validated scales informed by both literature and the lived experiences of participants. This community-based approach fostered buy-in from sites to survey their communities and ensured the measures were relevant to the community, promoting participation and meaningful data collection. We were mindful of preserving the integrity of the psychometric properties of the scales and, where possible, used original scales. We discuss our rationale for cases where scales were adapted to the study context in the Survey Development section and report on internal consistency estimates in the Measures section to support their use in this study.

## Methods

### Program Description

The dissemination of Headspace was a part of the Help@Hand project, a state-wide innovation project in California that aimed to understand how DMHIs fit within the public mental health system of care. The project explored how technology could be used to reach people who are likely to go either unserved or underserved by traditional mental health care. The project was funded and directed by multiple participating sites in California and overseen by the California Mental Health Service Authority.

To be eligible to enroll in the Headspace program, participants had to enter a zip code of the participating site where they lived, worked, or attended school at the time of enrollment. Participants did not have to be new users to Headspace, but they could not have an existing paid subscription at the time of enrollment. Participants enrolled via a specific web page launched by each site. After enrolling, participants had free access to Headspace up until the end of the program (see Table 1 for the different end dates of the programs across sites). Participants could enroll at any time during the program.

**Table 1:** Implementation launch and end dates of the Headspace Program in the 4 sites (Los Angeles County, San Mateo County, City of Berkeley, and Santa Barbara County). The first program launched in April 2020, and the last program ended in September 2023.

Site	Launch date	End date	Length of program
Los Angeles County	April 2020	February 2023	2 y and 11 mo
San Mateo County	September 2020	September 2021	1 y and 1 mo
City of Berkeley	October 2021	September 2023	2 y
Santa Barbara County	October 2021	September 2023	2 y

The Headspace program launch and end dates differed by site (Table 1), but the first program launched in April 2020 by Los Angeles County, and the last programs were launched in October 2021 by the City of Berkeley and Santa Barbara County.

### Headspace Description

Headspace is a commercially available meditation app that aims to improve mental wellness. The app offers meditation and mindfulness exercises in both audio and video format. Its content is organized into 4 categories: meditate, sleep, move, and focus.

- Meditate offers a library of single meditations and courses consisting of a series of meditations that vary in length and topic. For example, it offers meditation exercises and courses focused on reducing anxiety and stress, as well as on improving mood and productivity. It also offers a beginner’s course for users who are new to meditation.
- Sleep has guided sessions, meditations, and soundscapes intended for nighttime and to help with relaxation and falling asleep.
- Move has low-impact workout videos, such as yoga exercises, ranging from 10 to 30 minutes. The workouts are focused on incorporating mindfulness during movement.
- Focus offers music and meditation playlists that are intended to help focus during work.

The content is accessible through a mobile app, available on Android and Apple iOS, or the Headspace website, and the content is available in English, Spanish, French, German, and Portuguese.

Headspace was selected by the Help@Hand project after a vetting and approval process intended to assess the extent to which it met Help@Hand’s needs (eg, by considering how Headspace addressed privacy and security of their product and whether content was offered in multiple languages). It was able to meet the necessary specifications for use in this project and was available in languages other than English. Its popularity and reputation were also attractive. Headspace has more than 70 million subscribers worldwide [24] and is one of the most downloaded mindfulness apps in the world [25]. As Headspace, together with the meditation app Calm, represents the majority (approximately 90%) of users of DMHIs [4], capturing characteristics of the use of Headspace can be valuable in contributing to a better understanding of DMHI use.

### Survey Development

A community-based participatory approach was used to engage community members in the survey development. The research team initiated a work group with 9 key individuals from the 4 participating sites; the group met 5 times for 1 to 2 hours between January and July 2021. These key individuals included mental health advocates and project and program managers of behavioral health programs and site departments. During the sessions, work group members discussed and made decisions related to the survey method, instruments, and recruitment strategies. Members were given activities to provide feedback on the survey items both during and after the sessions.

Work group members provided multiple rounds of feedback on the draft surveys created by the research team. For example, concerns were raised about the tone of particular survey items and questions that may feel intrusive to participants. In response to this feedback, we supplemented 2 validated scales with additional survey items to balance positively worded items while maintaining items from validated scales that captured the necessary information. One item was added to the scale to measure loneliness: “How often do you feel connected with others?” Similarly, 1 item was added to the scale to measure distress: “During the last 30 days, about how often did you feel hopeful?” These additional items were included to enhance content balance but were not used in the calculation of loneliness and distress scores, respectively.

In addition, some survey items aimed at measuring mental health stigma were removed. The initial survey included measures of internalized stigma, resilience, and mental health treatment stigma based on key individual feedback from a 2019 in-person work group focused on stigma [26]. Following community members’ input, the mental health treatment stigma measure was replaced with perceived stigma and stigma resistance measures.

**Figure 1:**
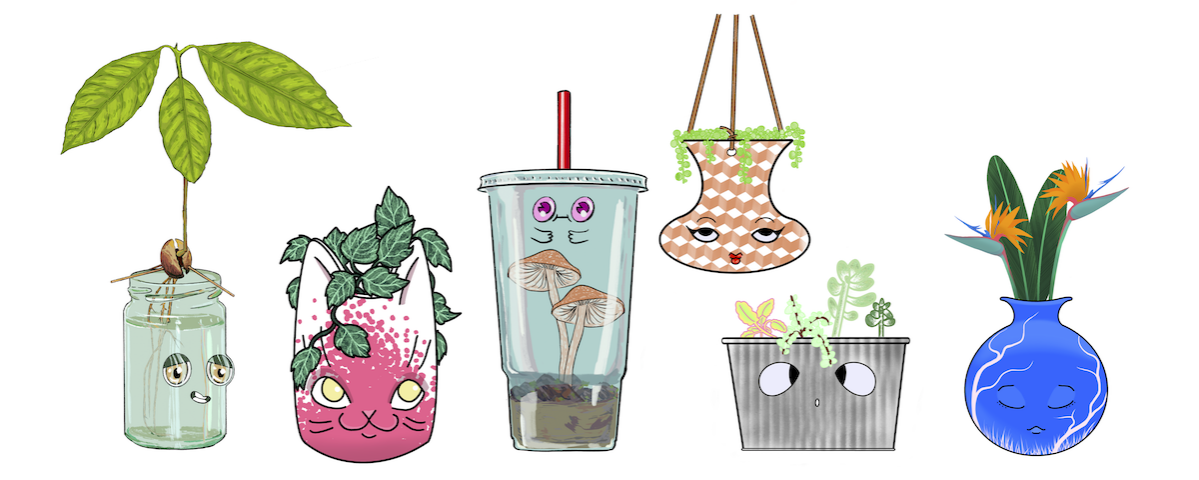
One of the art pieces created by community members to include in recruitment emails.

In addition, work group members vocalized the importance of highlighting wellness and well-being, while also being representative of the communities to whom the survey would be sent. Work group members recommended commissioning artwork from community members with lived mental health needs experiences, who were familiar with the diverse communities and could approach the task from a recovery perspective. Four art pieces were created by community members that incorporated themes of peer support, resilience, and multiformity. These art pieces were included in the emails sent out to participants to complete the survey (Figure 1 shows one of the art pieces).

The survey included questions related to people’s use of Headspace and experience, their use of mental health resources, and their health and well-being. On the basis of participants’ answers regarding their use of Headspace, different follow-up questions were asked (see Multimedia Appendix 1 for the survey instrument and the Measures section for a description of the survey measures).

### Ethical Considerations

The study was approved by the University of California, Irvine, Institutional Review Board (20195406). The survey included a study information sheet that was reviewed and approved by the institutional review board. Respondents were able to download a copy of the sheet. All research data collected were stored securely and confidentially on a password-protected secure server. Program participants were made aware when signing up for the innovation program that their email address would be shared with the research team and that they would be contacted to take part in a survey. Completing or not completing the survey did not affect people’s access to Headspace. Further details regarding the survey study are reported following the Checklist for Reporting Results of Internet e-Surveys guidelines [27] in Multimedia Appendix 2.

### Study Design and Procedure

This paper reports on survey data collected between July 1, 2021, and October 21, 2022.

After obtaining institutional review board and key individual approvals, the survey was finalized and deployed on July 1, 2021. Everyone in the participating sites who had signed up for Headspace up until that point were sent a survey. After that, the survey was sent once per week to all new people who had signed up in the past week. Participants were sent up to 5 email reminders (sent once every 3 days) to complete the survey. Once participants completed a survey, their email address was removed from the reminder email list so they did not receive any further reminders.

The research team was granted access to a dashboard which contained email addresses of participants who had signed up for the Headspace program via their site. All enrolled participants were sent an email to complete a web-based survey on their use of Headspace and experience, their use of other mental health resources, and their health and well-being. The survey was distributed via REDCap (Research Electronic Data Capture, version 15.5.0; Vanderbilt University) [28,29], a secure, web-based software platform designed to support data capture for research studies. Participants had to self-report to be aged ≥18 years to be eligible to complete the survey but did not need to have used Headspace as we also wanted to be able consider “nonuse” within the program, that is, people who signed up for the program but did not use Headspace. The survey could be completed on a computer, mobile phone, or tablet; was available in English and Spanish; took approximately 20 minutes to complete; and completing the survey was voluntary and uncompensated.

The survey was sent to 92,311 people and received by 92,261 people (50 emails bounced back). A total of 3399 participants started the survey, resulting in a response rate of 3.68% (3399/92,261). A total of 2725 participants completed the survey (completion rate=2.95%). Survey responses were anonymous and not linked to respondents’ email addresses. Only 1.03% (28/2725) of survey respondents completed the survey in Spanish; the other respondents completed the survey in English.

### Measures

#### Overview

A description of each survey measure is given below. We primarily focus on dichotomous measures as these have been validated using standard cutoff points. Further details about the measures are included in Multimedia Appendix 3.

#### Length of Use of Headspace

Respondents were asked when they signed up for the Headspace program. Respondents could choose from “Less than a week ago,” “2 weeks to 1 month ago,” “2 to 6 months ago,” “6 months to a year ago,” and “Longer than a year ago.”

#### Adoption of Headspace

Respondents were asked 1 multiple-choice question on whether they were using Headspace. Respondents could choose from the following answers: “Yes,” “No, but I did use it in the past,” or “No, and I never used it.” On the basis of respondents’ answers, we made a distinction between 3 types of users, which throughout the paper we call current users (who answered yes), past users (who used it in the past), and nonusers (who never used it).

#### Frequency of Use of Headspace

Respondents were asked 1 question on how frequently they used Headspace. While there is no gold standard on how to group frequency of use [30], past research has identified “active” users as individuals who use a mental health app daily [21], weekly or more [31], or monthly or more [32]. On the basis of the distribution of our sample, we grouped and dichotomized frequency of use into those who use it several times a week or more and those who use it less than that. Frequency of use was thus examined as a dichotomous measure as follows: 1 for “Daily/Several times a week” and 0 for “Several times a month/About once a month/I only used it once.”

#### Mental Health Challenges

Respondents were asked 1 multiple-choice question on whether they had experienced any mental health challenges (“Have you or do you experience mental health challenges?”). Respondents could indicate that they had “not experienced any challenges,” “had been diagnosed with a mental health condition,” “had not been diagnosed but were experiencing challenges,” or they could self-describe their experience. On the survey, it was explained that many different terms could be used to refer to these experiences, such as mental health challenge, emotional distress, and psychological disorder. The variable was operationalized as a dichotomous measure (1 for experiencing or having been diagnosed with a mental health challenge and 0 for not experiencing any challenges) in the analyses.

#### Distress

Psychological distress was measured using the Kessler Psychological Distress (K10) scale [33]. Respondents were asked to rate 10 statements related to how they had been feeling during the past 30 days (eg, “During the past 30 days, how often did you feel tired out for no good reason?”). The statements were rated on a 5-point Likert scale ranging from “None of the time” (score=1) to “All the time” (score=5), with a total sum score in the range of 10 to 50. A higher score indicates a higher level of psychological distress.

The internal consistency of this measure in this study was excellent (Cronbach α=0.91) [34]. The score was operationalized as a dichotomous measure in the analyses. In accordance with previous work [35-37], a cutoff score of >19 was used to categorize respondents as having distress.

#### Loneliness

Loneliness was measured using the 3-item Loneliness Scale [38], a shortened version of the University of California, Los Angeles Loneliness Scale [39]. Participants were asked to rate 3 statements related to loneliness on a 3-point Likert scale ranging from “Hardly ever” (score=1) to “Often” (score=3; eg, “How often do you feel left out?”), with a total sum score in the range of 3 to 9.

The internal consistency of this measure in this study was adequate (Cronbach α=0.78). The score was operationalized as a dichotomous measure in the analyses, and consistent with previous work [40], a cutoff score of ≥6 was used to categorize respondents experiencing loneliness.

#### Mental Health Stigma

Participants were asked to rate 4 statements related to mental health stigma (eg, “Being around people who don’t have mental health challenges makes me feel out of place or inadequate”) using a Likert scale ranging from “Strongly disagree” (score=1) to “Strongly agree” (score=5). The questions related to internalized stigma (1 item), perceived stigma (1 item), resilience (1 item), and stigma resistance (1 item). Items were taken from the Internalized Stigma of Mental Illness Inventory [41] for internalized stigma and stigma resistance, the Perceived Stigma subscale of the Depression Stigma Scale [42] for perceived stigma, and the Recovery Assessment Scale–Revised [43] for resilience. One item was used from each of these 4 subscales instead of all subscale items, based on input from the survey work group to keep the survey brief to mitigate response burden.

Items were chosen based on feedback to balance positively and negatively worded items, as well as key individual feedback from a 2019 in-person work group focused on stigma [26] and a subsequent factor analysis of responses from 6304 participants.

As the items measured different types of stigma and there was low internal consistency between stigma items (Cronbach α=0.58), the stigma items were analyzed separately and were not combined into a single score. This aligns with our community-based approach, which emphasized using measures that were both empirically grounded and meaningful to community members. Item scores were operationalized as dichotomous measures (1 for “Agree” and “Strongly agree” and 0 for “Neither agree nor disagree,” “Disagree,” or “Strongly Disagree”).

#### Mental Health Care Use

Four dichotomous (yes or no) response items from the California Health Interview Survey [44] were used to identify whether respondents had used online tools other than Headspace for problems related to mental health, whether they had connected online with people with similar mental health problems, whether they had used online tools to connect to a mental health professional, and whether they had seen a mental health professional in the past 12 months.

#### User Experience

Respondents who indicated they had used Headspace were asked questions related to their experience using Headspace. Participants were asked to rate 12 statements (eg, “I find Headspace useful in my daily life”) on a 5-point Likert scale ranging from “Strongly disagree” (score=1) to “Strongly agree” (score=5). Survey items were based on the Unified Theory of Acceptance and Use of Technology questionnaire [45], a questionnaire used to evaluate technology acceptance and adoption. The items showed high internal consistency (Cronbach α=0.91) and were combined as a single mean score that could range from 1 to 5. A higher score indicates a more positive experience with and opinion of Headspace. The score was operationalized as a dichotomous measure in the analyses, using a cutoff of >3 to constitute a positive experience [46].

### Reasons for Abandonment and Nonuse and Intention to Use Headspace

#### Overview

Respondents who indicated they were not currently using Headspace were asked to indicate their reasons for not using Headspace. Respondents were instructed to “select all that apply” from a list of 14 options or give an answer in their own words. The list of reasons was based on common barriers to engagement from the literature regarding mental health technology [47], as well as common answers given by community members in prior studies of the Help@Hand project. The full list of options is included in Multimedia Appendix 1.

Respondents were also asked a multiple-choice question on whether they intended to use Headspace in the future. Respondents could choose from “Yes,” “No,” and “I’m not sure.”

#### Digital Literacy

Respondents were asked to rate one statement related to digital literacy (“I am confident using technology to look up information”) taken from the Mental Health Literacy Scale [48] on a 5-point Likert scale ranging from “Strongly disagree” (score=1) to “Strongly agree” (score=5).

#### Demographic Measures

Respondents’ age was collected as a categorical variable to adhere to project requirements, with answer options “18 to 25,” “26 to 59” and “>60.” Respondents’ age was operationalized as mutually exclusive dummy variables (0-1) for 18 to 25, 26 to 59, and >60 years.

Respondents’ gender identity was operationalized as mutually exclusive dummy variables (0-1) for man or male, woman or female, and another identity (the survey included options to select the following: transgender man; transgender woman; genderqueer, gender nonconforming, or nonbinary; questioning or unsure of gender; or they could self-describe their identity).

Respondents’ race or ethnic identity was operationalized as mutually exclusive dummy variables (0-1) for non-Hispanic White, Hispanic or Latinx, Asian, Black or African American and another identity (the survey included options to select “American Indian, Native American, or Native Alaskan”; "Native Hawaiian or other Pacific Islander”; “Two or more races”; or they could self-describe their identity).

We first report on demographics and mental health symptoms of people who signed up. In the Results section, we explore the relationship between continued use of Headspace and demographics, prevalence of mental health problems, mental health care use, frequency of use of Headspace, user experience with Headspace, and mental health stigma. Finally, we report on respondents’ reasons for abandonment and nonuse of the app.

### Participants

The majority (2238/2725, 82.13%) of survey respondents were aged between 26 and 59 years. In total, 288 (10.57%) respondents were aged between 18 and 25 years, and 199 (7.3%) were aged ≥60 years; 1983 (72.77%) respondents identified as woman or female. Of 2725 respondents, 1246 (45.72%) respondents identified as non-Hispanic White, 496 (18.2%) identified as Hispanic or Latinx, 421 (15.45%) identified as Asian, and 119 (4.37%) identified as Black or African American. In total, 2003 (N=2725, 73.5%) 7respondents indicated they experienced mental health challenges; 1462 (N=2725, 53.65%) respondents were likely to experience distress, and approximately half (1469/2725, 53.9%) of the respondents scored high on loneliness. The majority (2575/2725, 94.5%) of survey respondents used Headspace in English, 1.47% (40/2725) used it in Spanish, 0.88% (24/2725) used it in German, and 0.15% (4/2725) used the app in French. Further demographic characteristics per user group are shown in Table 2. On the basis of respondents’ answers on whether they were using Headspace, we made a distinction between 3 user groups:

1. Current users; respondents who indicated they were using Headspace at the time of the survey
2. Past users; respondents who indicated they were not using Headspace anymore at the time of the survey but had used it in the past
3. Nonusers; respondents who indicated they never used Headspace

Table 3 shows the demographics for the sites, based on county census data [49]. When comparing demographics of survey respondents with the county data, our survey sample had a higher proportion of women or female respondents, a lower proportion of Hispanic or Latinx respondents, and individuals with higher levels of education.

**Table 2:** Demographic characteristics of survey respondents overall and by user group.

Demographics			Current users (n=2076), n (%)		Past users (n=570), n (%)		Nonusers (n=79), n (%)		Total (N=2725), n (%)
**Age (y)**									
	18-25	226 (10.9)		55 (9.6)		7 (8.9)		288 (10.6)	
	26-59	1717 (82.7)		464 (81.4)		57 (72.2)		2238 (82.1)	
	>60	133 (6.4)		51 (8.9)		15 (19)		199 (7.3)	
**Gender^a^**									
	Men	470 (22.6)		117 (20.5)		12 (15.2)		599 (22.0)	
	Women	1513 (72.9)		413 (72.5)		57 (72.2)		1983 (72.8)	
	Other gender identity	93 (4.5)		40 (7.0)		10 (12.7)		143 (5.2)	
**Race or ethnicity^a^**									
	Non-Hispanic White	965 (46.5)		243 (42.6)		38 (48.1)		1246 (45.7)	
	Hispanic or Latinx	384 (18.5)		100 (17.5)		14 (17.7)		498 (18.3)	
	Asian	296 (14.3)		113 (19.8)		12 (15.2)		421 (15.4)	
	Black or African American	92 (4.4)		27 (4.7)		0 (0)		119 (4.4)	
	Other race or ethnic identity	341 (16.4)		87 (15.3)		15 (19.0)		443 (16.3)	
**Highest education level**									
	Bachelor’s degree	923 (44.5)		258 (45.3)		30 (38)		1211 (44.4)	
	Graduate or professional degree	747 (36)		228 (40)		29 (36.7)		1004 (36.8)	
	Some college with no degree	179 (8.6)		35 (6.1)		7 (8.9)		221 (8.1)	
	Associates degree	103 (5.0)		19 (3.3)		4 (5.1)		126 (4.6)	
	High school or less than high school	65 (3.1)		13 (2.3)		1 (1.3)		79 (2.9)	
**Language they used Headspace in**									
	English	2015 (97.1)		560 (98.2)		N/A^b^		2575 (94.5)	
	Spanish	36 (1.7)		4 (0.7)		N/A		40 (1.5)	
	German or French^c^	22 (1.1)		6 (1.1)		N/A		28 (1)	

^a^See Multimedia Appendix 1 for all gender and race or ethnicity options.

^b^N/A: not applicable.

^c^Proportions of German and French users are merged given the small number of respondents in each category.

**Table 3:** Comparison of survey sample with county population sociodemographic characteristics. Our survey sample had a slightly higher proportion of women or female respondents, a lower proportion of Hispanic or Latinx respondents, and individuals with higher levels of education.

Demographics		Survey sample, n (%)	Aggregated county census data of Los Angeles, San Mateo, and Santa Barbara and City of Berkeley (%)
**Gender**			
	Woman or female	1983 (72.77)	50.3
**Race and ethnicity**			
	Non-Hispanic White	1246 (45.72)	39.3
	Hispanic, Latino, Latina, or Latinx	498 (18.28)	33.1
	Asian	421 (15.45)	18.8
	Black or African American	119 (4.37)	5.4
**Highest education level**			
	Bachelor’s degree or higher	2215 (81.28)	48.8

### Data Analysis

We analyzed the survey data using descriptive statistics (such as frequency counts) and inferential statistics. For each of the measures, we report proportions, means, and SD by user group. Chi-square tests were used to analyze differences between user groups in terms of age, gender, and race or ethnicity.

Logistic regressions were conducted for each outcome to control for site, length of use of Headspace, and covariates (age was added a covariate as there were significant differences in age between user groups) and to ensure that length of use of Headspace and any variations between sites did not affect the results. We report odds ratios (ORs) and 95% CIs. The analyses were performed using statistical software R (version 4.2.2) [50].

Total scores on loneliness, distress, and user experience with Headspace were only computed for respondents that completed ≥50% of the items on these respective scales. For respondents that completed ≥50% of the scale items, any missing data were imputed using mean imputation. Approximately 2% of participants had missing data imputed; <1% had a missing total score.

## Results

### Overview

Throughout this section, we report on the number of people who provided a particular survey answer and the total number of people who responded to the survey question.

Table 4 shows the number of respondents who were still using Headspace, were no longer using Headspace, and indicated they never used Headspace at the time of the survey. Approximately half of the respondents (1445/2725, 53.03%) had signed up for the Headspace program over a year ago. In total, 879 (32.26%) respondents had signed up between 2 months to a year ago and 398 (14.61%) had signed up <2 months ago.

**Table 4:** The number of people who were still using Headspace (current users), were no longer using Headspace (past users), and had never used Headspace (nonusers) at the time of completing the survey (N=2725).

User group	Sample, n (%)
Current users	2076 (76.18)
Past users	570 (20.921)
Nonusers	79 (2.9)

### Differences in Adoption by Demographics

There was a significant association between age and adoption of using Headspace (*χ*^2^_4_=21.1; *P*<.001): older people (ie, aged ≥60 y) were more likely to have never used Headspace (15/79, 19%) than be a current user (133/2076, 6.41%) or past user (51/570, 8.9%; Table 5).

Although there was no association between adoption and the proportion of women or female respondents, or men or male respondents (*χ*^2^_2_=1.9; *P*=.37), there was a significant association between adoption and the proportion of people who identified with another gender identity or did not answer the question: nonusers were more likely to identify with another gender identity or not disclose their gender identity (10/79, 13%) than current users (93/2076, 4.48%) and past users (40/570, 7%; *χ*^2^_4_=16.6; *P*=.002). There were no significant race or ethnicity differences in adoption (*χ*^2^_8_=15.1; *P*=.06).

**Table 5:** Demographics by adoption of Headspace. People aged ≥60 years were more likely to be nonusers, and nonusers were more likely to identify with another gender identity or not disclose their gender identity.

	Age^a^ (y), n (%)				Gender^b^, n (%)				Race or ethnicity, n (%)				
	18-25	26-59	>60	Male		Female	Other identity	Non-Hispanic White		Latinx	Asian	Black	Other race or identity
Current users	226 (10.9)	1717 (82.7)	133 (6.4)	22.6		1513 (72.9)	93 (4.4)	965 (46.5)		384 (18.5)	296 (14.3)	92 (4.4)	339 (16.3)
Past users	55 (9.6)	464 (81.4)	51 (8.9)	117 (20.5)		413 (72.5)	70 (7)	243 (42.6)		100 (17.5)	113 (19.8)	27 (4.7)	87 (15.3)
Nonusers	7 (8.9)	57 (72.2)	15 (19)	12 (15.2)		57 (72.2)	10 (12.7)	38 (48.1)		14 (17.7)	12 (15.2)	0 (0)	15 (19)

^a^*P*<.001.

^b^*P*<.01.

### Mental Health

**Figure 2:**
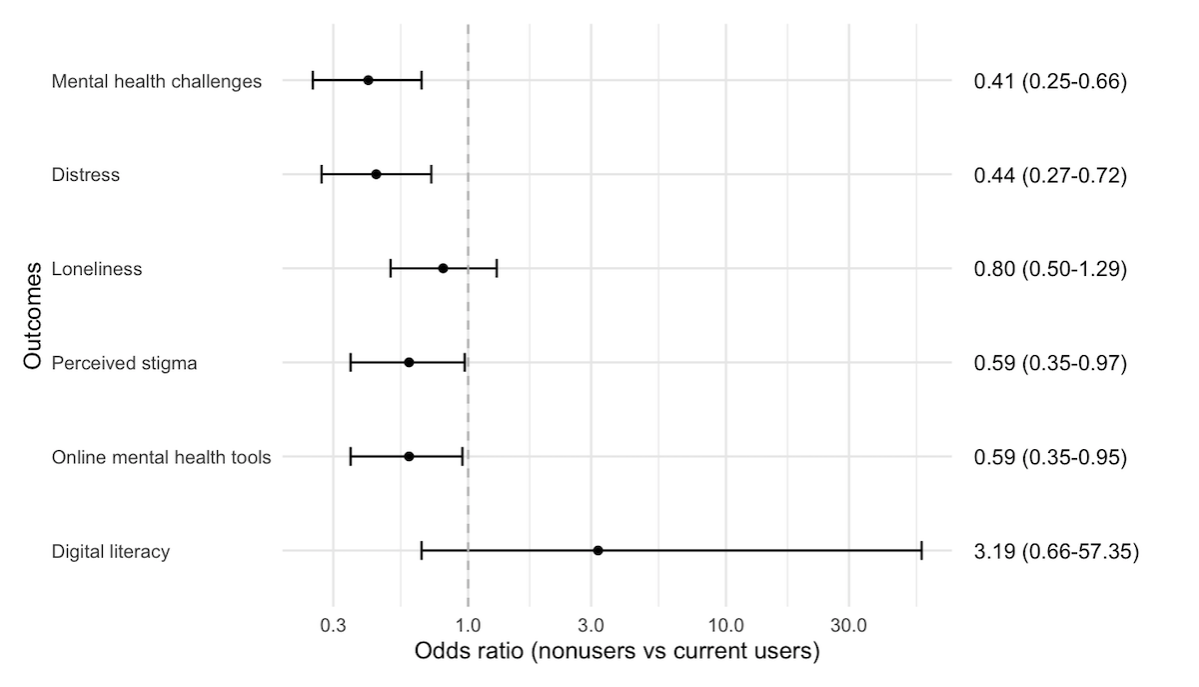
Odds ratios and 95% CIs for outcomes among current users compared with nonusers. Current users were more likely to experience mental health challenges and distress and make more use of online tools than nonusers.

**Figure 3:**
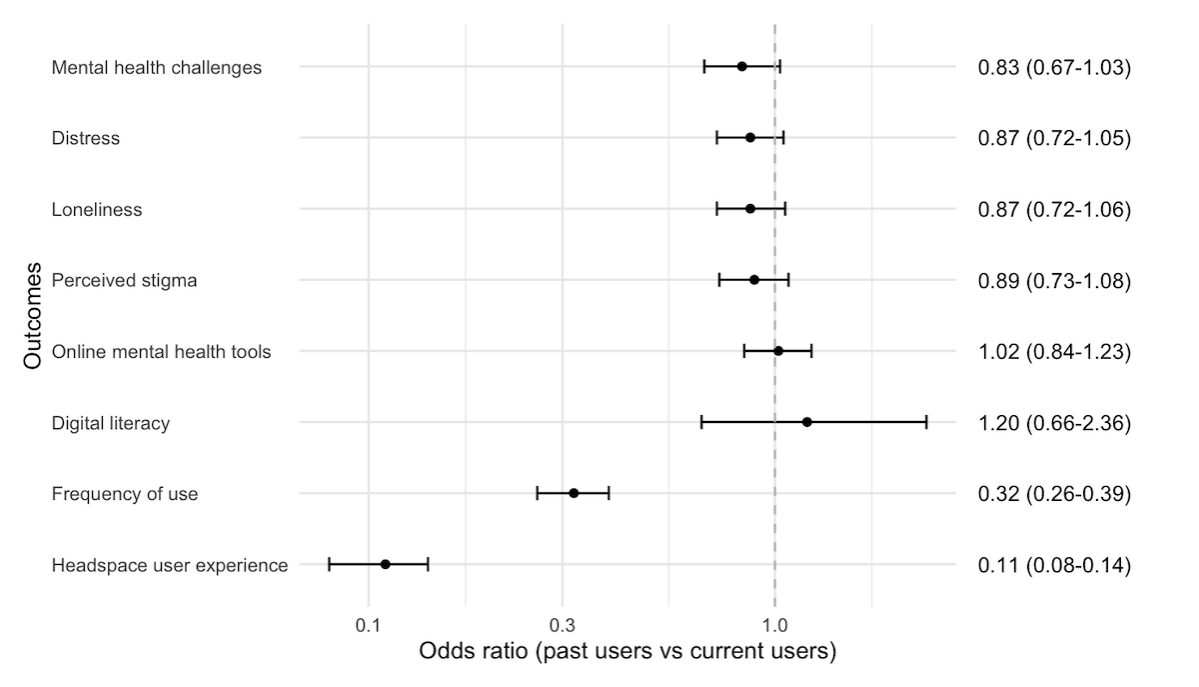
Odds ratios and 95% CIs for outcomes among current users compared with past users. Current users were more likely to rate Headspace high on user experience than past users.

Approximately 73.5% (2003/2725) of respondents indicated they currently experienced mental health challenges. Current users of Headspace were more likely to report having mental health challenges (1560/2076, 75.14%) than nonusers (40/79, 51%; OR 0.41, 95% CI 0.25-0.66, *P*<.001). Respondents who reported to experience distress were more likely to be current users (1184/2076, 57.03%) than nonusers (26/79, 33%; OR 0.44, 95% CI 0.27-0.72, *P*=.001). Figures 2 and 3 show the ORs and 95% CIs for outcomes among current users of Headspace compared with nonusers and past users, respectively.

Approximately 48.92% (1333/2725) of respondents scored high on loneliness, with no significant differences in loneliness between user groups (see Table S1 in Multimedia Appendix 4 for ORs).

### Mental Health Stigma

Respondents who agreed that most people believe that having mental health challenges is a sign of personal weakness (a perceived stigma measure) were more likely to be current users (904/2076, 43.55%) than nonusers (24/79, 30%; OR 0.59, 95% CI 0.35-0.97, *P*=.04). There were no differences on the other stigma items between users (see Table S2 in Multimedia Appendix 4 for ORs).

### Mental Health Care Use

Respondents who had made use of online mental health tools were more likely to be current users (900/2076, 43.35%) than nonusers (23/79, 29%; OR 0.59, 95% CI 0.35-0.95, *P*=.04). Respondents who had connected with online with people that had mental health concerns were also more likely to be current users (609/2076, 29.34%) than nonusers (14/79, 18%; OR 0.52, 95% CI 0.26-0.94, *P*=.04).

There were no significant differences in use of professional mental health services or in using online tools to be referred to professional mental health services between user groups (see Table S1 in Multimedia Appendix 4 for ORs).

### Digital Literacy

Most respondents (2620/2725, 96.15%) felt confident using technology to look up information, and there were no differences in digital literacy between user groups (see Table S2 in Multimedia Appendix 4 for ORs).

### Frequency of Use of Headspace

When thinking about what best describes their past use of Headspace, current users indicated they used Headspace more frequently than past users did before they stopped using it (OR 0.32, 95% CI 0.26-0.39, *P*<.001). Approximately 63.1% (1310/2076) of current users indicated they used Headspace daily or several times a week, compared with 33.4% (190/570) of past users.

### Headspace User Experience

Overall, experience with Headspace was positive (mean 4.10, SD 0.68), with current users more likely to report a positive Headspace user experience (mean 4.24, SD 0.58) than past users (mean 3.60, SD 0.77; OR 0.11, 95% CI 0.08-0.14, *P*<.001).

Only a third of respondents (901/2725, 33.06%) indicated they could get help from others with using Headspace if they had any difficulties, and this was true for current users (703/2076, 33.86%), past users (190/570, 33.86%), and nonusers (8/97, 8%).

### Reasons for Not Using Headspace and Intention to Use Headspace

The most common reasons for not using Headspace anymore were that past users were already using other strategies and resources to support their mental health (198/570, 34.7%), they just wanted to try Headspace out (167/570, 29.3%), they no longer needed Headspace (73/570, 12.8%), or they felt Headspace was not useful (46/570, 8.1%).

The most common reasons for not using Headspace at all were that nonusers were already using other strategies and resources to support their mental health (19/79, 24%), they thought Headspace would not be useful (10/79, 18%), they could not find the time to use it (5/79, 6%), or they only wanted to use traditional mental health services (5/79, 6%).

Past users who were not currently using Headspace were more likely to indicate they intended to use Headspace in the future at 51.6% (294/570) compared with only 20% (19/97) of nonusers.

## Discussion

The aim of this paper was to describe characteristics of people who decided to use, stop using, or not use Headspace within the context of a wide-scale deployment of Headspace for free to community members.

### Demographics and Use of Headspace

Similar to other studies assessing meditation app users [51,52], most survey respondents were women or female and non-Hispanic White. Respondents’ gender and racial or ethnic background was not related to adoption or continued use of Headspace in our study. This is in contrast to previous studies that found that females were more likely than other genders to engage with DMHIs [53] and use meditation [54].

However, age was found to be related to use. Older respondents (ie, those aged >60 y) were more likely to have not used Headspace. This is consistent with past research on DMHIs. Older adults can experience challenges with technology [55] and may be less comfortable using digital tools, which can impact DMHI use [56,57]. Such users might need additional support both to start using a DMHI and sustain long-term use. This relates to the increasing use of digital navigators to support these processes [58]. One evaluation of the impact of digital care navigation in routine care found a large increase in registration rates, especially when a digital navigator reached out on the day of referral [59]. In public health models, navigation might need to focus specifically on the types of users who need the most help, such as older adults.

### Mental Health Symptoms and Use of Headspace

More than half of the respondents reported experiencing mental health challenges, distress, and loneliness, and respondents who experienced mental health challenges and distress were more likely to be current users than nonusers. Presence of mental health symptoms has been reported as a motivator to use DMHIs [53,60,61]. Therefore, it is possible that those with greater mental health concerns may be more likely to continue to use Headspace, potentially due to expected benefits. Indeed, a recent metareview showed that app-delivered mindfulness interventions can significantly improve perceived stress, anxiety, and depression [7]. These findings suggest a potential pattern worth exploring in a longitudinal study, as people may be more motivated to continue using an intervention if it has potential benefits for mental health challenges they are experiencing [47].

### Headspace Experience and Use of Other Resources to Support Mental Health

Respondents overall rated Headspace highly on user experience, with current users indicating a more positive experience than past users. Prior studies on Headspace showed that a positive attitude toward use of the app enabled the use of Headspace [62].

Despite a positive experience with Headspace, 20.92% (570/2725) of respondents indicated they had already stopped using Headspace by the time they were surveyed. This is consistent with past work that has shown that nearly 1 in 4 people stop using DMHIs after only 1 use [63]. Given that such use is likely not an adequate dose of the intervention, supporting sustained use may be important to drive interventions impacts. In our study, the most common reasons for not using or abandoning Headspace were that respondents were already using other strategies to support their mental health or no longer needed Headspace. Other work has highlighted that a positive impact on one’s mental health can drive sustained use [32]; however, at some point when people have received sufficient benefits they are likely to stop using a DMHI. This type of “happy abandonment,” where people no longer need the intervention was more common in our sample than those who did not find Headspace useful (73/570, 12.8% vs 46/570, 8.1%). Most studies do not receive information for those who stop using a DMHI, and although “happy abandonment” has been posited elsewhere [32], our study is one of the first of its kind to be able to quantify it in a sample of users.

People’s use of other strategies and resources further highlights that although apps are often evaluated in isolation, people use a combination of various tools to support their health and well-being [64]. Each tool can have its own function within an individual’s life, and rather than understanding each tool in isolation, it may be worthwhile to understand this ecosystem of tools. Future studies can explore how Headspace fits into existing resources people are already using, what motivates them to adopt or discontinue use, and how these resources complement each other. It is also important to better understand individuals’ reasons behind switching between different resources, and the strengths and limitations of mobile apps in addressing more serious mental health challenges.

Current users were more likely to have made use of other online tools besides Headspace for their mental health, with almost half of current users having used another online tool. Past research has shown that increasing familiarity with DMHIs can impact user perceptions of DMHIs [65], and a past positive experience with digital health tools can increase likelihood to adopt other DMHIs in the future [66-68]. Our findings support this notion, suggesting that past experience and familiarity with digital tools may not only increase initial adoption but also facilitate continued use of apps such as Headspace. Notably, current users also used the app more frequently than other user groups, further underscoring the importance of early engagement in supporting continued use.

### Need for Support

Only a third of respondents reported they had access to help if they had difficulties using the app, with no differences between current and past users. Though overall experience with Headspace was positive in our study, this lack of access highlights an important potential barrier, as technical issues are one of the most common barriers to using DMHIs [47]. People may be enthusiastic about a technology but be dissuaded from using it if they are experiencing technical barriers. This is another opportunity for digital navigators as discussed earlier. The efficiency model of human support [69] posits 5 potential failure points to using DMHIs including usability, engagement, fit, knowledge, and implementation. Overcoming technical barriers and usability issues has been found in other studies to be a main activity supporters engage in [69]. Studies should further explore what types of help people are interested in and design support programs to address these issues.

### Implications and Future Directions

#### Overview

Mindfulness meditation apps can be a useful mental health resource to provide to communities. Our findings identified user characteristics associated with continued app use in the context of an innovation program. While the results of this study are exploratory, they can help generate hypotheses for future research and inform outreach strategies, especially within programs that aim to use DMHIs as a public mental health intervention. We outline several implications that may be important to consider.

#### Understand the Relationship Between Use and Mental Health Symptoms

Current users were more likely to experience mental health challenges and distress, which is a potential indication that people who need support are more likely to use it when options are made available. Future studies should seek to further elucidate the relationship between the use of Headspace and mental health symptoms. For example, it may be worthwhile to explore if continued use and engagement is associated with any improvement in mental health symptoms over time, and whether the severity of distress impacts perceived usefulness and ability to use meditation apps.

#### Engagement Metrics Alone Are Only Part of the Story

Despite continued concerns regarding poor engagement rates in real-world contexts of DMHI, future work should look beyond user engagement and understand reasons for not using an intervention. In our study, the number of people who stopped using Headspace because they no longer needed it outnumbered those who stopped using it because they did not find it useful. We should not assume that nonuse equals bad use. It is also possible that people might return to these tools over time if they found them useful. Indeed, we found that past users of Headspace were more likely to report an expectation of using Headspace in the future than those who never started using it (nonusers). Future studies could explore how and why people return to these tools over time, especially if longitudinal data are available.

#### Consider Ecologies of Care

Respondents used other mental health resources beyond Headspace, and the most common reason for not using Headspace was that they already had other strategies in place to support their mental health. Tools are often not used in isolation but exist within an ecosystem of tools used to manage one’s mental health and well-being [64]. Furthermore, DMHIs may fit into a continuum of care that could include formal (ie, treatment) and nonformal (ie, social support and religious communities). Future work could further investigate how Headspace fits into people’s existing toolbox of digital and non–digital mental health resources.

### Limitations

Our study had considerable strengths, including the large sample and real-world deployment. It is worth considering these strengths in light of the study’s limitations as well. A common issue with survey studies evaluating app use is reporting and response bias [51]. The use of Headspace was self-reported, and most of our survey respondents indicated they were current users, who may have had more positive opinions on Headspace and had more prevalent mental health challenges. Indeed, despite our large sample, we only received responses from 3% of the 92,311 users identified in the deployment. Our study sample differed from the overall population demographics of the participating sites, with more respondents having higher levels of education, a greater proportion identifying as women or female, and fewer identifying as Hispanic or Latinx. However, we cannot conclude if this was reflective of those who started using Headspace or merely those who completed our survey. Community members who did not sign up at all may have experienced additional barriers, such as comfort or experience with apps and access to required resources to use apps. Nevertheless, prior studies found similar trends with women [53,70] and the highly educated [71] being more likely to access DMHIs. Future program efforts may benefit from expanding outreach strategies to reach underrepresented groups, such as by featuring men in promotional materials [72] or developing culturally and linguistically appropriate materials to reach diverse communities.

Furthermore, our survey was launched after the program had started. Launch dates differed by site, and respondents had signed up for the program at different time points at the time of the survey launch. While adding length of use as a control variable did not affect any of the reported relationships between continued use and dependent variables, individuals who had signed up and stopped using Headspace a while ago at the time of administering the survey, never used Headspace, or had a bad experience may have been less likely to participate. In addition, the survey was cross-sectional, which limits our ability to track changes in outcomes over time. To address this limitation, follow-up surveys have been distributed to respondents to enable longitudinal analysis, which will be the focus of a future study.

Finally, the demographic survey items were designed according to project requirements: as required by California Code of Regulations (Cal. Code Regs. Tit. 9, § 3580.010), projects categorized and funded as innovative projects in California must adhere to specific data collection and reporting guidelines [73]. This restriction limited certain types of analyses. Age, for example, was asked as a categorical rather than continuous variable, which limits our ability to make conclusions on any correlations between age and use of Headspace. Some of the other measures may not conform to typical research measures; for example, validated stigma item scales were shortened based on stakeholder feedback to mitigate response burden and remove survey items that did not resonate with the community. However, our community-based participatory approach to survey development incorporated and centered the views of partners from the sites as well as those with lived-experience. Collecting data in real-world deployments often requires balancing the views and priorities of multiple invested parties.

### Conclusions

To our knowledge, this is the first paper reporting the characteristics of app use with a large sample of potential and naturalistic users with the mindfulness app Headspace. Results showed that people who were still using Headspace were more likely to experience mental health challenges and distress and had made more use of other digital mental health resources (ie, online tools and connecting with people online) than people who were not using Headspace. In addition, current users of Headspace used the app more frequently than people who abandoned the app and rated the app higher on usefulness. The most common reasons for not using Headspace were that people were already using other strategies to support their mental health, could not find the time to use Headspace, or did not think Headspace would be useful. These results can be used to inform future outreach strategies and implementations of future digital mental health programs.

## Appendix

Multimedia Appendix 1 The survey instrument.

Multimedia Appendix 2 Checklist for Reporting Results of Internet e-Surveys.

Multimedia Appendix 3 The survey measures as defined in the analyses.

Multimedia Appendix 4 Logistic regression tables showing odds ratios and 95% CIs for all measures.

## Abbreviations

DMHI: digital mental health intervention
OR: odds ratio
REDCap: Research Electronic Data Capture

## Data Availability

The survey instrument is included in Multimedia Appendix 1. The data collected and analyzed during the study are not publicly available as it was not a funding requirement, and study participants did not consent for their data to be shared publicly.
