# Supplementary material for: Characteristics Associated With the Use of the Mindfulness Meditation App Headspace in a Large Public Health Deployment: Cross-Sectional Survey Study
Source: JMIR Form Res. 2025 Aug 22;9:e73457. doi: 10.2196/73457 (PMC12413571; doi:10.2196/73457)
Supplement: Multimedia Appendix 1 [file formative_v9i1e73457_app1.docx]

**Survey Questions**

| **Q#** | **Question** |
| --- | --- |
| 0. | What language do you prefer to take the survey? / ¿En qué idioma desea completar la encuesta?   - English / Inglés - Spanish / Español |
| 1. | How old are you? (Select one answer.)   - 0-17 - 18-25 - 26-59 - 60+ |
| 2. | *If over 18:* **The first part of the survey will ask about your technology use in general.**  Which of the following do you use? (Select all that apply.)   - Desktop or laptop computer   - My own   - Shared or public - Smartphone   - My own   - Shared or public - Tablet (e.g., iPad)   - My own   - Shared or public - Mobile phone or cell phone but not a smartphone   - My own   - Shared or public - I don't use any of these - I prefer not to answer |
|  | *If you have a smartphone OR mobile phone:* Do you have concerns about the storage space on your phone when downloading apps? (Select one answer.)   - Yes - No - I prefer not to answer |
| 3. | Do you have a mobile data plan? (Select one answer.)  A mobile data plan allows you to access the internet without WiFi.   - Yes - No - I’m not sure. - I prefer not to answer |
| 4. | Please rate the extent to which you agree or disagree, using a scale from **Strongly disagree (1)** to **Strongly agree (5)**.   \|  \| Strongly disagree-1 \| Somewhat disagree-2 \| Neither agree nor disagree-3 \| Somewhat agree-4 \| Strongly agree-5 \| \| --- \| --- \| --- \| --- \| --- \| --- \| \| I am confident using technology to look up information. \|  \|  \|  \|  \|  \| |
| 5. | **The next part of the survey will ask about Headspace.**  About how long ago did you sign up for Headspace? (Select one answer.)   - Less than a week ago - 2 weeks to 1 month ago - 2 months to 6 months ago - 6 months to a year ago - Longer than year ago |
| 6. | Are you currently using Headspace? (Select one answer.)   - Yes - No, but I did use it in the past - No, and I never used it |
| 7. | *If previous Q "yes" or "no, but I did use it in the past":* What best describes how often you use or used Headspace? (Select one answer.)   - Daily - Several times a week - Several times a month - About once a month - I only used it once - Other (please explain): __________________________________ |
| 8. | *If never used is selected:* Why did you not use Headspace? (Select all that apply.)   - I thought Headspace would be too difficult to use or take up too much of my time. - I don’t have enough data on my data plan or space on my phone to use Headspace. - I didn't have consistent access to a smartphone or internet connection to use Headspace. - I thought Headspace wouldn't be useful. - I wouldn’t have been able to get help from others if I had difficulties using Headspace. - I was worried what other people might think of me if I used Headspace. - I was concerned that my data would not be private on Headspace. - I was concerned Headspace wouldn’t understand my culture. - Headspace was not in a language that I want to use. - I wanted to handle the problem myself. - I was using other strategies to support my mental health and wellness that work well for me. - I only wanted to use traditional mental health and wellness services. - Other (please explain): ________________________________________________ |
| 9. | *If NOT currently using Headspace:* Why did you stop using or take a break from Headspace? (Select all that apply.)   - Headspace was too difficult to use or took up too much of my time. - I lost access to Headspace and couldn't figure out how to get it back. - I don’t have enough data on my data plan or enough space on my phone or tablet to use Headspace. - I didn't have consistent access to a smartphone or internet connection to use Headspace. - Headspace was not useful. - I was worried what other people might think of me if they knew I used Headspace. - I was concerned that my data was not private on Headspace. - Headspace didn’t understand my culture. - Headspace was not in a language that I want to use. - I felt like I no longer needed Headspace or had reached my goals using Headspace. - I was using other strategies to support my mental health and wellness that work better for me than Headspace. - I wanted to use only traditional mental health and wellness services. - I wanted to handle the problem myself. - I just wanted to try Headspace out. - Other (please explain): ________________________________________________ |
| 10. | *If yes to stopped using Headspace or never used Headspace was selected:* Do you intend to use Headspace in the future? (Select one answer.)   - Yes - No - I'm not sure - I prefer not to answer |
| 11. | *If never is NOT selected:* Did you share your Headspace app with anyone? (Select one answer.)   - Yes - No - I prefer not to answer |
| 11. | Who did you share your Headspace app with? (Select all that apply.)   - Family member - Spouse or partner - Friend - Co-worker - Neighbor - Other (please explain): _______ |
| 13. | *If never is NOT selected:* Which language did you mainly use Headspace in? (Select one answer.)   - Spanish - English - French - German - Portuguese |
| 14. | Please rate the extent to which you agree or disagree, using a scale from **Strongly disagree (1)** to **Strongly agree (5)**.   \|  \| Strongly disagree-1 \| Somewhat disagree-2 \| Neither agree nor disagree-3 \| Somewhat agree-4 \| Strongly agree-5 \| Does not apply \| \| --- \| --- \| --- \| --- \| --- \| --- \| --- \| \| I would recommend Headspace to someone like myself. \|  \|  \|  \|  \|  \|  \| \| It is easy to fit Headspace into my everyday life and activities. \|  \|  \|  \|  \|  \|  \| \| Headspace meets my mental health and wellness needs. \|  \|  \|  \|  \|  \|  \| \| I find Headspace useful in my daily life. \|  \|  \|  \|  \|  \|  \| \| Using Headspace improves my mental health. \|  \|  \|  \|  \|  \|  \| \| Using Headspace improves my wellness. \|  \|  \|  \|  \|  \|  \| \| Using Headspace makes me feel like I have more support when I am feeling down. \|  \|  \|  \|  \|  \|  \| \| Please select "Somewhat  disagree - 2" for this statement.  This is a data quality check. \|  \|  \|  \|  \|  \|  \| \| Using Headspace makes me feel like I have more support when I am feeling stressed. \|  \|  \|  \|  \|  \|  \| \| Using Headspace helps me feel more confident seeking mental health and wellness services (such as therapy or counseling). \|  \|  \|  \|  \|  \|  \| \| I currently use Headspace to support my wellness in between therapy sessions. \|  \|  \|  \|  \|  \|  \| \| I think Headspace is easy to use. \|  \|  \|  \|  \|  \|  \| \| I can get help from others when I have difficulties using Headspace. \|  \|  \|  \|  \|  \|  \| \| Headspace values and respects cultural differences. \|  \|  \|  \|  \|  \|  \| |
| 15. | **The next set of questions ask about your well-being, mental wellness, and mental health.**  Many people have experienced or are currently experiencing a mental health challenge that impacts their day to day life, such as their family, relationships, or work. There are many different terms used to refer to those experiences—some terms include “emotional distress,” “mental illness”, “mental health challenge”, “psychological disorder,” “mental health condition,” "mentally unwell", etc.  Have you or do experience mental health challenges? (Select one answer.)   - Yes, I have been diagnosed with a mental health challenge - Yes, I experience mental health challenges but have not been diagnosed by a professional - No, I don't experience mental health challenge - Other (Please explain): _______________ - I prefer not to answer |
| 16. | The next set of statements are going to ask you about how you have been feeling during the past 30 days. For each statement, please rate how often you have been feeling that way over the past 30 days, on a scale from **None of the time (1) to All of the time (5).**  During the **last 30 days**, about how often did you feel...   \|  \| None of the time-1 \| A little of the time-2 \| Some of the time-3 \| Most of the time-4 \| All of the time-5 \| \| --- \| --- \| --- \| --- \| --- \| --- \| \| ...tired out for no good reason? \|  \|  \|  \|  \|  \| \| ...nervous? \|  \|  \|  \|  \|  \| \| ...so nervous that nothing could calm you down? \|  \|  \|  \|  \|  \| \| ...hopeless? \|  \|  \|  \|  \|  \| \| ...restless or fidgety? \|  \|  \|  \|  \|  \| \| ...so restless you could not sit still? \|  \|  \|  \|  \|  \| \| ...depressed? \|  \|  \|  \|  \|  \| \| ...that everything was an effort? \|  \|  \|  \|  \|  \| \| ...so sad that nothing could cheer you up? \|  \|  \|  \|  \|  \| \| ...worthless? \|  \|  \|  \|  \|  \| \| ...hopeful? \|  \|  \|  \|  \|  \| |
| 17. | The next set of questions are about mental health stigma. Stigma can cause people to feel badly for something that is out of their control. Keep in mind, these statements don't represent our views of mental illness.  Throughout this section, the term "mental health challenges" will be used. However, there are other terms that could be used, such as mental illness, psychological disorder, or mental health condition  Please rate the extent to which you agree or disagree, using a scale from **Strongly disagree (1)** to **Strongly agree (5)**.   \|  \| Strongly disagree-1 \| Somewhat disagree-2 \| Neither agree nor disagree-3 \| Somewhat agree-4 \| Strongly agree-5 \| Does not apply \| \| --- \| --- \| --- \| --- \| --- \| --- \| --- \| \| Being around people who don’t have mental health challenges  makes me feel out of place or inadequate \|  \|  \|  \|  \|  \|  \| \| Most people believe that having mental health challenges is a sign of personal weakness. \|  \|  \|  \|  \|  \|  \| \| I know when to ask for help. \|  \|  \|  \|  \|  \|  \| \| In general, I am able to live life the way I want to. \|  \|  \|  \|  \|  \|  \| |
| 18. | **The next set of questions will ask you about resources you use to support your well-being, mental wellness, and mental health**.  While these questions use the phrase "problems with your mental health, emotions, nerves," there are many other ways to refer to challenges or concerns about your wellness, emotional health, well-being, or mental health.  In the past **12 months**, have you tried to get help from an online tool (including mobile apps or texting services) **other than Headspace** for problems with your mental health, emotions, nerves? (Select one answer.)   - Yes - No - I prefer not to answer |
| 19. | In the past **12 months**, have you connected online with people that have mental health concerns through methods such as social media, blogs, and online forums? (Select one answer.)  *Includes online forums or closed social media groups on specific issues, doing hashtag searches on social media, or following people with similar health conditions*   - - Yes   - No   - I prefer not to answer |
| 20. | In the past **12 months**, have you used online tools to find, be referred to, contact, or connect with a mental health professional? (Select one answer.)  *For example, by texting, on-line messaging, video chat, or through a mental health or health-related mobile app or website*   - - Yes   - No   - I prefer not to answer |
| 21. | In the past **12 months** have you seen a professional, such as a counselor, psychiatrist, or social worker for problems with your mental health, emotions, nerves? (Select one answer.)   - Yes - No - I prefer not to answer |
| 22. | **The next set of questions ask about your feelings of connection and isolation.**  People may feel lonely or isolated even if they have people in their lives. For many, the pandemic has worsened feelings of loneliness because of the changes to our everyday lives. Some people may not be able to participate in typical in-person activities.  The next questions are about how you feel about different aspects of your life. For each one, please answer how often you feel that way.  How often do you feel...   \|  \| Hardly ever \| Some of the time \| Often \| \| --- \| --- \| --- \| --- \| \| ...that you lack companionship? \|  \|  \|  \| \| ...left out? \|  \|  \|  \| \| ...isolated from others? \|  \|  \|  \| \| ...connected with others? \|  \|  \|  \| |
| 23. | **The last part of the survey is going to ask about your background and demographics**.  Some of these questions may feel personal. Please know we ask everyone these questions to make sure we represent the viewpoints of your community. You can skip any questions you don’t feel comfortable answering.  What is the zip code where you live? ______ |
| 24. | What is your gender? (Select one answer.)   - Man / male - Woman / female - Transgender man - Transgender woman - Genderqueer / Gender non-conforming / Non-binary - Questioning or unsure of gender - I prefer to self-identify: __________ - I prefer not to answer |
| 25. | What is your sexuality? (Select one answer.)   - Gay or Lesbian - Heterosexual or Straight - Bisexual - Pansexual - Queer - Asexual - Questioning or unsure of sexuality - I prefer to self-identify: __________ - I prefer not to answer |
| 26. | What best describes your race/ethnicity? (Select one answer.)   - American Indian/Native American/ Native Alaskan - Asian - Black or African American - Hispanic/Latino/a/x - Native Hawaiian or other Pacific Islander - Non-Hispanic White/Caucasian - Prefer to self identify:____ - Two or more races - I prefer not to answer |
| 27. | What is your yearly household income? (Select one answer.)   - Under $20,000 - $20,000 - $39,999 - $40,000 - $59,999 - $60,000 - $79,999 - $80,000 - $99,999 - $100,000 - $149,999 - $150,000 or above - I don't know. - I prefer not to answer. |
| 28. | What is the highest level of education you have completed? (Select one answer.)   - Less than high school (no diploma or GED) - High school graduate (or GED) - Some college with no degree - Associate’s degree - Bachelor’s degree - Graduate or professional degree - Other (Please explain):_____ - I prefer not to answer. |
| 29. | Have you had a change in your employment because of the coronavirus/COVID-19 (Select all that apply.)   - Lost my job - Had my hours reduced - Same job and hours, but working less due to children at home/other caregiving responsibilities - Working from home/remotely - my choice - Working from home/remotely - required by employment/government mandate - Other (Please explain)____ - No change in my employment - I prefer not to answer. |
| 30. | What else would you like to share with us?  ___________________________ |
