# Supplementary material for: Characteristics Associated With the Use of the Mindfulness Meditation App Headspace in a Large Public Health Deployment: Cross-Sectional Survey Study
Source: JMIR Form Res. 2025 Aug 22;9:e73457. doi: 10.2196/73457 (PMC12413571; doi:10.2196/73457)
Supplement: Multimedia Appendix 3 [file formative_v9i1e73457_app3.docx]

**Survey Measures Defined**

|  | **Survey Measure** | **Operationalization** |
| --- | --- | --- |
| **Mental Health** |  |  |
| Mental Health Challenges | Have you experienced or do you experience mental health challenges? | 0=”No, I don’t experience a mental health challenge”  1=”Yes, I have been diagnosed with a mental health challenge” or “Yes, I experience mental health challenges but have not been diagnosed by a professional” |
| Distress | Kessler Distress Scale | 0=negative screen (0-19)  1=positive screen (20-50) |
| Loneliness | Three-Item Loneliness Scale | 0=negative screen (0-5)  1=positive screen (6-9) |
| **Knowledge and Attitudes** |  |  |
| Internalized Stigma | Being around people who don’t have mental health challenges makes me feel out of place or inadequate. | 0=“Neither agree nor disagree,” “Disagree,” or “Strongly Disagree”  1=“Strongly agree” or “Agree” |
| Perceived Stigma | Most people believe that having mental health challenges is a sign of personal weakness. | 0=“Neither agree nor disagree,” “Disagree,” or “Strongly Disagree”  1=“Strongly agree” or “Agree” |
| Treatment-seeking Stigma | I know when to ask for help. | 0=“Neither agree nor disagree,” “Disagree,” or “Strongly Disagree”  1=“Strongly agree” or “Agree” |
| Stigma Resistance | In general, I am able to live life the way I want to. | 0=“Neither agree nor disagree,” “Disagree,” or “Strongly Disagree”  1=“Strongly agree” or “Agree” |
| Digital Literacy | I am confident using technology to look up information. | 0=“Neither agree nor disagree,” “Disagree,” or “Strongly Disagree”  1=“Strongly agree” or “Agree” |
| **Use of Mental Health Resources** |  |  |
| Online Tool | In the past 12 months, have you tried to get help from an online tool (including mobile apps or texting services) other than Headspace for problems with your mental health, emotions, nerves? | 0=no  1=yes |
| Connecting with people online | In the past 12 months, have you connected online with people that have mental health concerns through methods such as social media, blogs, and online forums? | 0=no  1=yes |
| Referral professional help | In the past 12 months, have you used online tools to find, be referred to, contact, or connect with a mental health professional? | 0=no  1=yes |
| Professional help | In the past 12 months have you seen a professional, such as a counselor, psychiatrist, or social worker for problems with your mental health, emotions, nerves? | 0=no  1=yes |
| **Use and User Experience** |  |  |
| User Experience | UTAUT | 0=“Neither agree nor disagree,” “Disagree,” or “Strongly Disagree”  1=“Strongly agree” or “Agree” |
| Frequency of Use | What best describes how often you use or used Headspace? | 0=”Several times a month”, “About once a month”, or “I only used it once”  1=”Daily” or “Several times a week” |
| **Reasons for Non-Use** | Why did you not use Headspace? | 1 = I thought Headspace would be too difficult to use or take up too much of my time.  2 = I don’t have enough data on my data plan or space on my phone to use Headspace.  3 = I didn't have consistent access to a smartphone or internet connection to use Headspace.  4 = I thought Headspace wouldn't be useful.  5 = I wouldn’t have been able to get help from others if I had difficulties using Headspace.  6 = I was worried what other people might think of me if I used Headspace.  7 = I was concerned that my data would not be private on Headspace.  8 = I was concerned Headspace wouldn’t understand my culture.  9 = Headspace was not in a language that I want to use.  10 = I wanted to handle the problem myself.  11 = I was using other strategies to support my mental health and wellness that work well for me.  12 = I only wanted to use traditional mental health and wellness services.  13 =“Other” |
| **Reasons for Stopping or Taking a Break from Headspace** | Why did you stop using or take a break from Headspace? | 1 = Headspace was too difficult to use or took up too much of my time.  2 = I lost access to Headspace and couldn't figure out how to get it back.  3 = I don’t have enough data on my data plan or enough space on my phone or tablet to use Headspace.  4 = I didn't have consistent access to a smartphone or internet connection to use Headspace.  Headspace was not useful.  5 = I was worried what other people might think of me if they knew I used Headspace.  6 = I was concerned that my data was not private on Headspace.  7 = Headspace didn’t understand my culture.  8 = Headspace was not in a language that I want to use.  9 = I felt like I no longer needed Headspace or had reached my goals using Headspace.  10 = I was using other strategies to support my mental health and wellness that work better for me than Headspace.  11 = I wanted to use only traditional mental health and wellness services.  12 = I wanted to handle the problem myself.  13 = I just wanted to try Headspace out  14 =“Other” |
| **Covariates/Other** |  |  |
| Age | How old are you? | 1=18-25  2=26-59  3=60+ |
| Race/Ethnicity | What best describes your race/ethnicity? | 0=Non-Hispanic White  1=Hispanic/Latino/a/x  2=Asian  3=Black or African American  4=Two or more races  5=American Indian/Native American/Native Alaskan, Native Hawaiian or other Pacific Islander |
| Gender | What is your gender? | 0=”Man/male”  1=”Woman/female”  2= ”Transgender man”, ”Transgender woman”, “Genderqueer / Gender non-conforming / Non-binary”, “Questioning or unsure of gender” or “I prefer to self-identify” |
