# Supplementary material for: Characteristics Associated With the Use of the Mindfulness Meditation App Headspace in a Large Public Health Deployment: Cross-Sectional Survey Study
Source: JMIR Form Res. 2025 Aug 22;9:e73457. doi: 10.2196/73457 (PMC12413571; doi:10.2196/73457)
Supplement: Multimedia Appendix 4 [file formative_v9i1e73457_app4.docx]

**Table A1.** Logistic regressions of mental health challenges, distress, loneliness, and mental healthcare utilization.

|  | Mental Health Challenges | Distress | Loneliness | Online tools | Connecting with people online | Referral mental health professional | Use of professional mental health services |
| --- | --- | --- | --- | --- | --- | --- | --- |
| Abandoners | 0.83 [0.67, 1.03] | 0.87 [0.72, 1.05] | 0.87 [0.72, 1.06] | 1.02 [0.84, 1.23] | 1.12 [0.91, 1.38] | 0.91 [0.75, 1.10] | 0.87 [0.72, 1.05] |
| Non-Users | 0.41 [0.25, 0.66]*** | 0.44 [0.27, 0.72]** | 0.80 [0.50, 1.29] | 0.59 [ 0.35, 0.95]* | 0.52 [0.26, 0.94]* | 0.89 [0.54, 1.46] | 0.83 [0.52, 1.35] |
| City of Berkeley | 0.71 [0.51, 1.00]* | 0.80 [0.58, 1.09] | 0.66 [0.48, 0.89]** | 1.16 [0.86, 1.56] | 0.84 [0.60, 1.17] | 0.87 [0.64, 1.18] | 1.06 [0.79, 1.44] |
| San Mateo County | 0.80 [0.60, 1.07] | 0.59 [0.45, 0.77]*** | 0.77 [0.59, 1.01] | 1.14 [0.88, 1.49] | 1.04 [0.78, 1.39] | 0.81 [0.61, 1.07] | 1.11 [ 0.85, 1.46] |
| Santa Barbara County | 1.11 [0.66, 1.93] | 0.62 [0.39, 1.00]* | 0.68 [0.43, 1.07] | 0.92 [0.57, 1.45] | 0.73 [0.42, 1.25] | 0.91 [0.56, 1.47] | 1.41 [0.88, 2.28] |
| Age 18-25 | 1.89 [1.36, 2.68]*** | 1.91 [1.45, 2.53]*** | 1.70 [1.32, 2.21]*** | 1.02 [0.79, 1.32] | 1.44 [1.10, 1.87]** | 1.20 [0.93, 1.55] | 1.14 [ 0.89, 1.48] |
| Age 60+ | 0.34 [0.25, 0.46]*** | 0.34 [0.25, 0.47]*** | 0.68 [0.50, 0.93]* | 0.63 [0.46, 0.86]** | 0.50 [0.33, 0.72]*** | 0.35 [0.24, 0.50]*** | 0.43 [0.31, 0.59]*** |
| Signed up less than two months ago | 0.97 [0.79, 1.19] | 0.68 [0.56, 0.83]*** | 0.82 [0.69, 0.99]* | 1.03 [0.86, 1.24] | 0.94 [0.78, 1.15] | 1.00 [0.83, 1.20] | 1.04 [0.87, 1.24] |
| Signed up between two and six months ago | 0.98 [0.82, 1.17] | 1.02 [0.87, 1.20] | 0.99 [0.84, 1.15] | 0.95 [0.82, 1.11] | 0.93 [0.79, 1.10] | 1.00 [0.85, 1.17] | 1.16 [0.99, 1.35] |

**Note:** Table values are odds ratios with 95% CIs in brackets. Reference groups are: current users (for Type of user), Los Angeles County (for County/city), and age 26-59 (for Age).

*** *P* < .001.

** *P* < .01.

* *P* < .05.

**Table A2.** Logistic regressions of frequency of use, user experience, digital literacy, and mental health stigma.

|  | Freq. of Use | User  Experience | Digital Literacy (‘I am confident using technology to look up information.’) | Internalized Stigma (‘Being around people who don’t have mental health challenges makes me feel out of place or inadequate’) | Perceived Stigma (‘Most people believe that having mental health challenges is a sign of personal weakness.’) | Stigma Resilience (‘I know when to ask for help.’) | Stigma Resistance (‘In general, I am able to live life the way I want to.’) |
| --- | --- | --- | --- | --- | --- | --- | --- |
| Abandoners | 0.32 [0.26, 0.39]*** | 0.11 [0.08, 0.14]*** | 1.20 [0.66, 2.36] | 0.92 [0.74, 1.15] | 0.89 [0.73, 1.08] | 1.00 [0.76, 1.31] | 1.00 [0.76, 1.31] |
| Non-Users | - | - | 3.19 [0.66, 57.35] | 0.55 [0.27, 1.03] | 0.59 [0.35, 0.97]* | 1.75 [0.94, 3.10] | 0.93 [0.44, 1.77] |
| City of Berkeley | 1.31 [0.94, 1.82] | 0.68 [0.40, 1.22] | 0.71 [0.32, 1.79] | 1.04 [0.74, 1.45] | 0.63 [0.46, 0.85]** | 0.86 [0.56, 1.29] | 1.00 [0.67, 1.48] |
| San Mateo County | 0.80 [0.60, 1.06] | 1.49 [0.92, 2.49] | 0.45 [0.23, 0.92]* | 0.74 [0.53, 1.03] | 1.02 [0.78, 1.34] | 0.50 [0.31, 0.77]** | 0.59 [0.38, 0.90]* |
| Santa Barbara County | 0.78 [0.46, 1.31] | 0.56 [0.25, 1.36]* | 1.45 [0.45, 6.52] | 1.23 [0.72, 2.08] | 1.29 [0.81, 2.07] | 0.98 [0.47, 1.88] | 1.33 [0.73, 2.35] |
| Age 18-25 | 0.71 [0.54, 0.93] | 1.36 [0.82, 2.37] | 4.05 [1.23, 25.03] | 2.39 [1.84, 3.10]*** | 0.90 [0.70, 1.17] | 2.17 [1.59, 2.94]*** | 1.50 [1.09, 2.05]* |
| Age 60+ | 1.12 [0.81, 1.55] | 0.52 [0.33, 0.83]** | 0.29 [0.16, 0.53]*** | 0.38 [0.23, 0.60]*** | 1.15 [0.85, 1.56] | 0.82 [0.49, 1.29] | 0.93 [0.59, 1.42] |
| Signup_1 | 0.55 [0.44, 0.67]*** | 1.31 [0.88, 1.89] | 1.89 [1.15, 3.04]** | 1.05 [0.85, 1.29] | 0.86 [0.72, 1.03] | 1.10 [0.85, 1.43] | 0.92 [0.72, 1.17] |
| Signup_2 | 1.38 [1.17, 1.63]*** | 1.13 [0.84, 1.52] | 0.81 [0.51, 1.28] | 0.85 [0.71, 1.02] | 0.99 [0.85, 1.16] | 0.80 [0.65, 1.00]* | 0.96 [0.78, 1.19] |

**Note:** Table values are odds ratios with 95% CIs in brackets. Reference groups are: current users (for Type of user), Los Angeles County (for County/city), and age 26-59 (for Age).

*** *P* < .001.

** *P* < .01.

* *P* < .05.
